# Reprogramming histone modification patterns to coordinate gene expression in early zebrafish embryos

**DOI:** 10.1186/s12864-019-5611-7

**Published:** 2019-03-28

**Authors:** Wei Zhu, Xiaocui Xu, Xinxin Wang, Jiang Liu

**Affiliations:** 1 CAS Key Laboratory of Genome Sciences and Information, Beijing Institute of Genomics, Chinese Academy of Sciences, Beijing, 100101 China; 2 University of Chinese Academy of Sciences, Beijing, 100049 China

**Keywords:** Histone modification, Early embryo, Reprogramming, Epigenetics, Conservation

## Abstract

**Background:**

Multicellular organisms require precise gene regulation during ontogeny, and epigenetic modifications, such as DNA methylation and histone modification, facilitate this precise regulation. The conservative reprogramming patterns of DNA methylation in vertebrates have been well described. However, knowledge of how histone modifications are passed on from gametes to early embryos is limited, and whether histone modification reprogramming is conserved is not clear.

**Results:**

We profiled H3K4me3/H3K27me3 modifications in gametes and early embryos in zebrafish and found that the patterns in gene promoter regions have been largely set to either co-occupied or active states in gametes and then passed on to early embryos. Co-occupied states are partially maintained, while active states are largely restored to nearly match the sperm’s pattern prior to zygotic genome activation (ZGA). However, repressive H3K27me3 modifications in promoter regions are largely discarded in early embryos. Prior to ZGA, patterns of genes that initialize ZGA are converted to nonrepressive states to coordinate gene expression. Moreover, promoter peaks that mark stage-specific genes are hypermethylated, and histone modifications in these regions are erased independently of DNA methylation reprogramming. Furthermore, comparative analysis revealed that the functions of co-occupied and active genes passed on from gametes are conserved in vertebrates. Gene age preferences by co-occupied and active histone modifications are also confirmed in vertebrates.

**Conclusions:**

Our data provide fundamental resources for understanding H3K4me3/H3K27me3 modifications in early zebrafish embryos. The data also reveal that the reprogramming progress of histone modifications is conserved in vertebrates and coordinates with gene expression during ZGA.

**Electronic supplementary material:**

The online version of this article (10.1186/s12864-019-5611-7) contains supplementary material, which is available to authorized users.

## Background

Multicellular organisms have evolved numerous cell types that provide sophisticated biological functions and thus require delicate gene regulation mechanisms. Epigenetic modifications such as DNA methylation and histone alterations play vital roles in cell-type decisions. Histones are found in a wide range of eukaryotic organisms, and modifications of histones, such as methylation, acetylation and phosphorylation, are crucial epigenetic marks informing gene activities and chromatin states. These modifications are found around the transcription start site (TSS) of genes in diverse states, in the gene body and in the heterochromatin regions, and they even serve as a signature of DNA damage [1]. Of the currently known modifications, trimethylation of histone H3 lysine 4 (H3K4me3), which marks active genes, and trimethylation of histone H3 lysine 27 (H3K27me3), which marks repressed genes, are the most common modifications found in TSS regions. A combination of these two modifications marks genes as co-occupied or in a transcriptionally poised state, which is a characteristic of genes committed to cell differentiation [2].

The reprograming dynamics of DNA methylation (5mC) during early embryo development have been well illustrated in zebrafish [3, 4] and mice [5]. However, knowledge on histone modification reprogramming is limited. It has been reported for yeast and HeLa cells that histone H3 lysine 9 methylation in heterochromatic regions could be inherited by copying the modification pattern from nearby parental nucleosomes [6, 7]. Additionally, asymmetric histone segregation may play roles in maintaining pluripotency in Drosophila male germline stem cells [8]. These findings imply that there may be reprogramming principles to be followed in cell cycles. Meanwhile, chromatin in sperm is more densely packaged compared to that in oocytes. In contrast to mammalian sperm, where the majority of histones are replaced by protamine during spermatogenesis [9], abundant linker histone H1 variants have been detected in zebrafish sperm [10]. According to the above findings, histone modification reprogramming is highly possible in early embryos. Recent, studies [11, 12] have revealed the dynamics of broad H3K4me3 domains found in mouse oocytes but not in zebrafish. We wondered how the histone modifications are reprogrammed in zebrafish and whether the reprogramming principles are conserved in vertebrates as it is for DNA methylation.

The most challenging part of profiling histone modifications in early embryos is that chromatin immunoprecipitation (ChIP) requires large amounts of experimental materials, and early stage embryos are not affordable. Particularly for zebrafish, there is abundant yolk protein that may interfere with immunoprecipitation. Consequently, previously published studies largely focused on stages around ZGA (zygotic genome activation) where a relatively large number of cells are available. Hints from currently available results have indicated that in zebrafish, the competition between histone and gene transcription factors for binding to DNA regulates the onset of ZGA [13] and histone marks had long been established before ZGA [14, 15]. However, the process of transitioning histone modification status from gametes to zygotes is not clear. In this study, we optimized a ChIP method for early zebrafish embryos based on previous publications [16, 17] by adding extra washing steps that efficiently remove the yolks from the embryos and eggs [18]. We generated H3K4me3/H3K27me3 histone modification profiles for gametes and 2-cell, 16-cell, 128-cell and 1 K-cell stages with thousands of embryos or eggs. Together with public datasets for the embryo of the dome [19] and 48hpf [20] stages, we compared the histone modification landscapes between mouse and zebrafish. We found conservation in histone modification reprogramming and their preference in gene age.

## Results

### Overviews of histone modification dynamics in early zebrafish embryos

We profiled genome-wide H3K4me3 and H3K27me3 occupancy in zebrafish gametes and early embryos (including 2-cell, 16-cell, 128-cell and 1 K-cell stages) with ChIP-seq. For each stage, data from two replicates (Additional file 1: Figure S1a) were combined with equivalent read pairs. Peaks were called and then annotated around the TSS to identify associated genes. Compared with published ChIP-chip data for sperm [10] and the 1 K-cell stage [15], our data uncovered many more genes and covered the majority of genes found by previous studies (Additional file 1: Figure S1b). We noticed that the peak widths (Additional file 1: Figure S2) and CpG dinucleotide density (Additional file 1: Figure S3) in promoter peak regions are higher than those in distal peak regions for both H3K4me3 and H3K27me3.

**Fig. 1:**
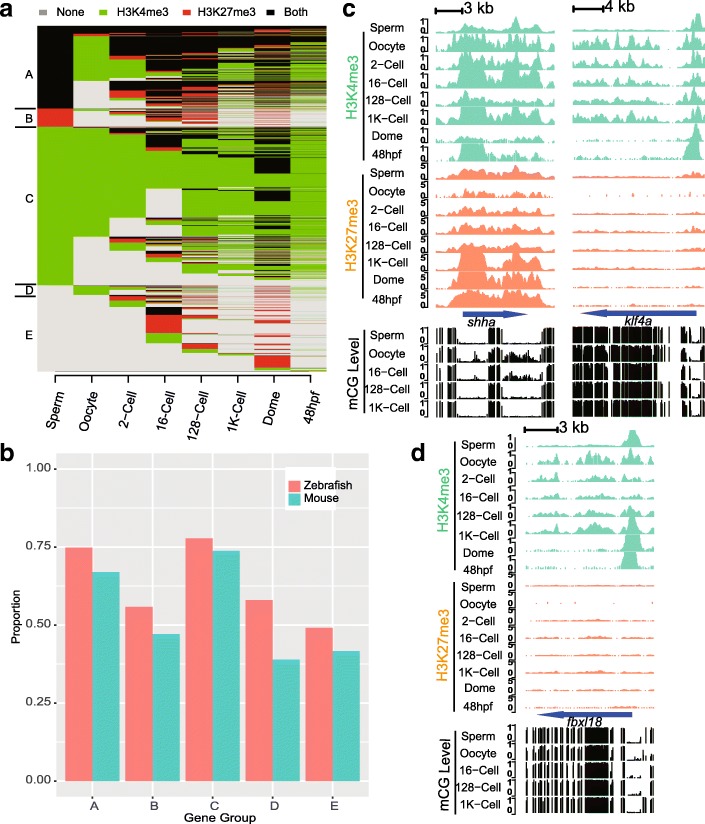
Overview of histone modification dynamics in zebrafish. **a** Histone modification dynamics for genes in zebrafish. **b** Proportion of homologous genes found in each gene group between zebrafish and mouse according to the ENSEMBL Compara database. **c** UCSC Genome Browser snapshots of histone modifications and DNA methylation for the *ssha* and *klf4a* genes. **d** UCSC Genome Browser snapshots of histone modifications and DNA methylation for the *fbxl18* gene

To provide an understanding of the global reprogramming progress of histone modifications in early zebrafish embryos, all genes marked by histone modifications were categorized into five groups (A-E) primarily according to their states in gametes (Fig. 1a). To our surprise, 23.9% of marked genes are in a co-occupied state in sperm (i.e., group A), and this proportion ranks highest among all of the investigated stages. Moreover, only 204 of those co-occupied genes, which are developmental regulators, e.g., *shha* (Fig. 1c), *sox2* (Additional file 1: Figure S4), *pou5f3* (Additional file 1: Figure S4) and *hox* clusters (Additional file 1: Figure S5), have a stable histone modification status throughout all of the investigated stages. Gene group C, which contains genes in an active state in sperm, represents nearly half (45.7%) of all marked genes. Another 22% of marked genes are embryo specific genes (group E). Repressed genes in sperm (group B) and oocyte-specific genes (group D) represent 5.3 and 2.8% of marked genes, respectively.

We also compared the histone modification dynamics in zebrafish with those of early mouse embryos (Additional file 1: Figure S6). The global patterns are approximately stabilized at the 2-cell stage, where the mouse zygotic genome initializes activation. Genes are divided into five groups depending on their states in gametes, and similar reprogramming characteristics are observed. In mouse, gene groups A to E represent 11.3, 15.9, 30.7, 30 and 12.1% of all marked genes, respectively. As in zebrafish, sperm is the main source of genes marked both by H3K4me3 and H3K27me3. For genes in group A, their co-occupancy is lost rapidly by conversion to an active state, except for 30 genes remain unchanged. The status of active genes (group C) is stabilized in the 2-cell stage. The largest difference between mouse and zebrafish is found in gene group D, which contains the oocyte-specific modified genes. Group D represents nearly as many genes as the active group (group C) in mouse, but only represents 2.8% in zebrafish. Furthermore, we examined the proportions of homologous genes in corresponding gene groups between zebrafish and mouse and found that the majority of genes in groups A and C are homologous (Fig. 1b).

Previous studies of DNA methylation have revealed larger methylation-level differences between gametes in mouse than in zebrafish. In our histone modification data, stage-wise dissimilarities (Additional files 2 and 3) also suggested that there are sperm-oocyte differences in histone modification status between mouse (0.66) and zebrafish (0.44). Taken together with the broad H3K4me3 domains reported in mouse oocytes but not in zebrafish oocytes, we speculate that the histone modification reprogramming processes in zebrafish and mouse share similarities but differ in detail.

#### Distinct reprograming patterns for genes in different histone modification states

Genes in different modification states undergo distinct reprogramming procedures. The co-occupied gene group (group A) undergoes extensive reprogramming progress in zebrafish compared to that group in mouse. For these genes, less than 10% (543 genes) of co-occupied genes are shared in sperm and oocyte, i.e., germline co-occupied genes. However, less than half of these germline co-occupied genes’ states are kept stable across the investigated stages, such as *shha* (Fig. 1c), *sox2* (Additional file 1: Figure S4), *pou5f3* (Additional file 1: Figure S4) and *hox* clusters (Additional file 1: Fig. S5). Sperm-specific co-occupied genes progressively lost their co-occupancy after the first zygotic cleavage, leaving 15% (770 genes) of them remaining in co-occupied states across all of the investigated stages. Notably, for those sperm-specific co-occupied genes that lost their co-occupancy, they tend to be converted to active states if they are in an active state in oocytes; otherwise they are converted to repressive states and then lose their repressive markers eventually. As a result of the above reprogramming, the final state in the early embryo is not similar to either the sperm or the oocyte, but instead shares features of both gametes. However, in mouse, based on the currently available data, the reprogramming procedure is straightforward, namely, H3K27me3 enrichments are removed from the corresponding co-occupied gene group after the first cell cleavage.

For the active gene group (group C), histone modifications are partially erased and then reprogrammed nearly but not completely to match the sperm’s pattern upon ZGA, similar to DNA methylation. In our data, we observed that H3K4me3 modifications of more than half of genes are first erased until the 16-cell stage and then restored around the 1 K-cell stage (Fig. 1d, *fbxl18*; Additional file 1: Figure S4, *blf*). Among these genes, gamete-shared genes, e.g., *gfm1* (Additional file 1: Figure S4) are immediately reestablished, while sperm-specific genes, e.g., *spef2* (Additional file 1: Figure S4) (3338 genes), undergo extensive reprogramming and are not restored,. A similar pattern is also observed in mouse for the corresponding gene group.

Repressed genes, including sperm-originated (group B) and oocyte-originated H3K27me3-marked genes, e.g., *klf4a* (Fig. 1c), which are germline H3K27me3-occupied genes that restrict the maternal-to-zygotic genome transition [21], are erased gradually at the ZGA time point,. Although there is a difference in the proportion of oocyte-specific and embryo-specific genes between mouse and zebrafish, these genes are found to lose their histone modifications independent of modification types in mouse and zebrafish.

#### Histone modification patterns are established prior to gene expression

**Fig. 2:**
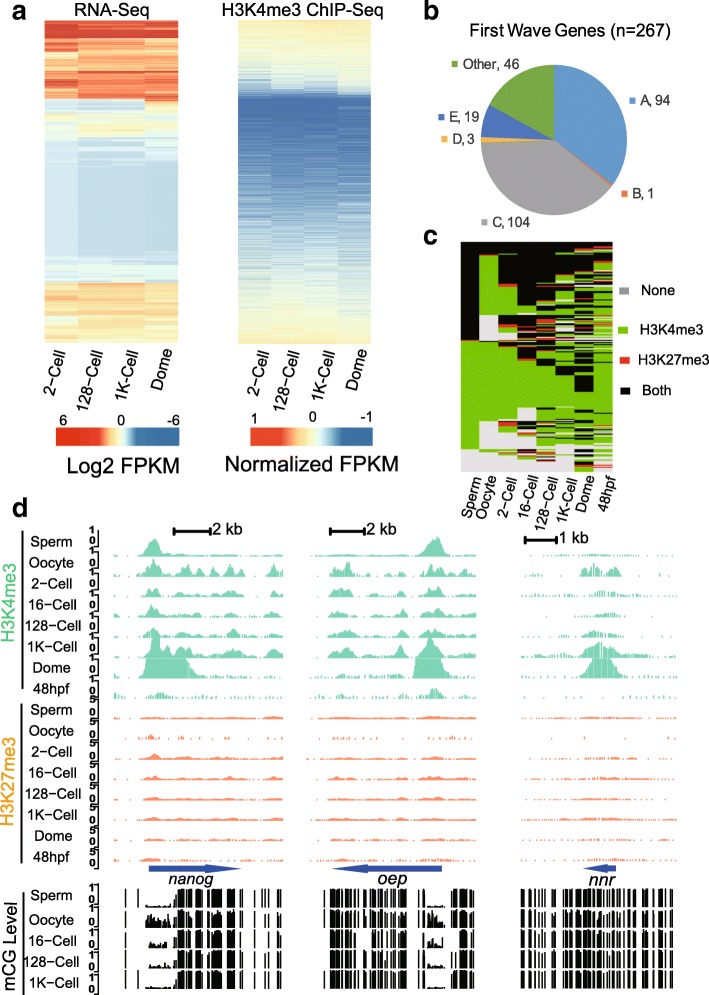
Histone modification patterns coordinate with gene expression prior to ZGA. **a** Heatmap of RNA-Seq abundance and H3K4me3 enrichments for the 2-cell, 128-cell, 1 K-cell and dome stages. **b** Proportion of the first wave of genes expressed during ZGA for each gene group. we demonstrate that group A and E represent majority of early expressed genes. **c** Histone modification states for the first wave of expressed genes **e** Example snapshots of histone modifications and DNA methylation for the first wave genes *nanog*, *oep* and *nnr*

Histone modifications could be indicators of gene expression activities. In our data, we found that gene expression and the gene histone modification intensity of H3K4me3 in promoter regions are globally positively correlated (Fig. 2a). Moreover, the correlation efficiency increased for H3K4me3 modifications as early embryo development proceeded (Additional file 1: Figure S7). However, for H3K27me3, there are weak but negative associations between histone modification intensity in promoter regions and gene expression (Additional file 1: Figure S7).

Prior to ZGA, gene expression levels are static, as they are mostly driven by maternal deposition, and expression levels increase upon the onset of ZGA [22]. To further characterize histone modification dynamics during ZGA, we examined histone modification patterns for genes that are expressed in the first wave in early ZGA of zebrafish embryos. Previously studies has uncovered a set of genes that expressed at the very beginning of ZGA [23]. Here we found that those early expressed genes largely consist of co-occupied and active gene groups (Fig. 2b) along with members from other groups. These early expression genes lack H3K27me3 modifications at the 1 K-cell stage (Additional file 1: Figure S1c). Histone modification patterns of key developmental regulators that are initialized at ZGA in zebrafish, including *pou5f3*, the *soxB* gene family and *nanog*, were examined, and we found that histone modification co-occupancy on *pou5f3*, *sox19b* and *sox2* (Additional file 1: Figure S4) are stable across the investigated stages. However, this result did not hold true for *nanog* (Fig. 2e), which was reported to induce 70% of the first wave gene s [23], H3K4me3 enrichment has long been established early from gametes and embryos, but H3K27me3 modifications are removed after the 2-cell stage to match the pattern of sperm and then are restored after ZGA (48hpf stage). Another example of an early expressed gene with loss of repressive markers during ZGA is *lft1* (Additional file 1: Figure S4). *lft1* is an inhibitor of the *nodal* signaling pathway, which is responsible for mesoderm induction and L-R axis development [24, 25]. H3K4me3 enrichment is accompanied by loss of H3K27me3 enrichment around ZGA. However, for those genes induced by *nanog*, e.g., *cldnb* (Additional file 1: Figure S4), *oep* and *nnr* (Fig. 2d), H3K4me3 enrichment is initialized at the 1 K-cell stage, and a clear peak emerged at the dome stage. Overall, histone modification patterns of genes expressed in the first wave are converted to co-occupied or active states prior to gene expression (Fig. 2c). These findings are in line with the results from published data demonstrating that histone modification patterns are established prior to ZGA [15].

#### Distinct DNA methylation patterns in distal and promoter peaks

**Fig. 3:**
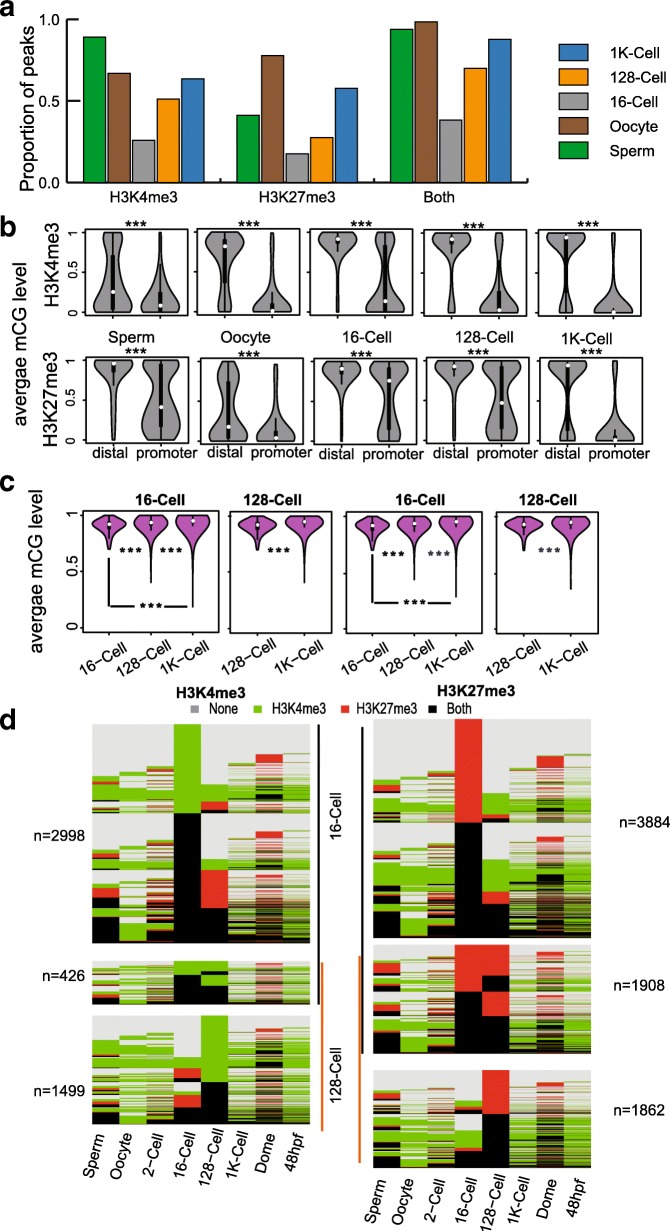
DNA methylation patterns in distal and promoter peaks. **a** Bar plot for proportion of peaks that overlap with hypomethylated regions in each stage. **b** Violin plot demonstrates the differences between distal and promoter peaks in each stage for H3K4me3 and H3K27me3. **c** Violin plot showing the DNA methylation pattern dynamics in hypermethylated promoter peaks found in 16-cell and 128-cell stages for H3K4me3 and H3K27me3. **d** Histone modification dynamics for hypermethylated promoter peak-associated genes found in the 16-cell and 128-cell stages. Note: ‘***’ denotes that there is a significant difference detected by the Wilcox test

It is known that the DNA methylome of early embryos undergoes extensive reprogramming. To investigate the relationship between DNA methylation and histone modification reprogramming in the early embryo, we first examined the correlations between DNA methylation levels and histone enrichments around TSS and found that there are weak but negative correlations between them for both H3K4me3 and H3K27me3 (Additional file 1: Figure S8a). We then checked the histone modification status of the DNA methyltransferase gene family (Additional file 1: Figure S8b) and found that there are no repressive H3K27me3 peaks found in their promoter regions before ZGA, which coincides with their expressions [22]. Additionally, we noticed that there are dynamics in the proportion of all peaks overlapping with hypomethylated regions. The proportion first decreased in early embryo stages and then gradually increased in later stages (Fig. 3a). After examination of the DNA methylation levels in distal and promoter peak regions, we concluded that hypermethylated (average methylation level not less than 0.7) promoter peaks are responsible for the reduction in the proportion of hypomethylated peaks in early embryos, apart from significant differences in DNA methylation levels between distal and promoter peaks (Fig. 3b). Moreover, we also observed that unlike zebrafish embryos, there are minimal hypermethylated peaks in mouse, and DNA methylation levels decreased from gametes to early embryos for both distal and promoter peaks (Additional file 1: Figure S9). We wondered if the disappearance of these hypermethylated promoter peaks is related to DNA methylation reprogramming. We examined DNA methylation dynamics in hypermethylated promoter peaks found in the 16-cell and 128-cell stages. To our surprise, DNA methylation levels in those hypermethylated promoter peaks do not decrease in later stages (Fig. 3c). By further checking the histone modification dynamics for those genes with hypermethylated promoter peaks, we found that the histone modification patterns of associated genes are distinct to adjacent stages (Fig. 3d). Therefore, these hypermethylated promoter peaks mark stage-specific genes and the reprogramming process is independent of DNA methylation reprogramming.

## Functional conservation of active and co-occupied genes

**Fig. 4:**
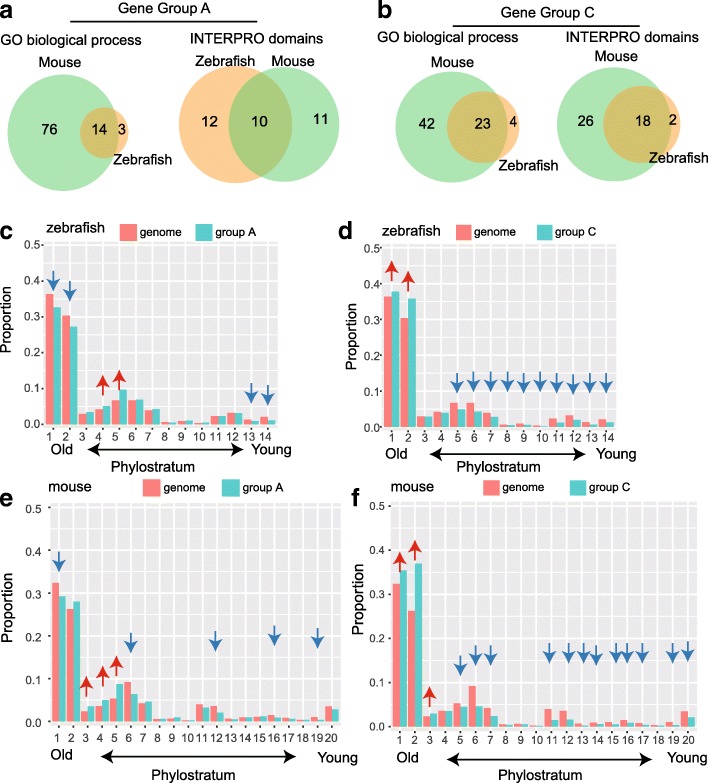
Functional conservation and gene age preference in histone modifications. **a** Overlap of enriched GO categories and INTERPRO domains in co-occupied genes between zebrafish and mouse. **b** Overlap of enriched GO categories and INTERPRO domains in active genes between zebrafish and mouse. **c** Proportion of each phylostratum in the co-occupied gene group and the whole genome for zebrafish. **d** Proportion of each phylostratum in the active gene group and the whole genome for zebrafish. **e** Proportion of each phylostratum in the co-occupied genes and the whole genome for mouse. **f** Proportion of each phylostratum in the active genes and whole genome for mouse. **Note**: arrows above each phylostratum indicating significant differences in the proportion of each phylostratum between indicated gene groups and the whole genome, where red arrows denote the proportion is significantly higher and blue arrows denote the proportion is significantly lower in the indicated groups compared to the whole genome

We performed gene ontology (GO) and protein domain enrichment analysis for each gene group in zebrafish and mouse to see if there are conservations in gene functions (Additional files 4, 5, 6, and 7). After comparing the enriched items between zebrafish and mouse, we found that the majority of categories enriched in zebrafish were found in mouse, including both gene function and protein domains (Fig. 4a and b).

Co-occupied gene groups in zebrafish and mouse were found to be key developmental regulators (Additional file 1: Figure S10a), including homeo-box, *myc*-type and forkhead-type transcription factors, and these genes are responsible for transcriptional regulation, cell differentiation, ion transport and nervous system development (Additional file 1: Figure S10b). Such features of co-occupied genes suggested crucial roles in embryogenesis as previously reported in embryonic stem cells [2], human sperm [26] and other animals [27]. Genes in the active group encoded protein structures characterized as zinc finger, WD40 and helicase domains (Additional file 1: Figure S10c). Active gene groups are functionally enriched for various GO categories (Additional file 1: Figure S10d) related to fundamental cellular processes, including protein transport and posttranscriptional modification, cell cycle/division and cellular organelle processing. We did not find any conservation for other gene groups between mouse and zebrafish.

Considering this analysis, together with histone modification reprogramming patterns, we proposed that histone modifications in promoter regions are reprogrammed according to the genes’ biological functions. Genes maintaining fundamental cell life are instantly marked by active H3K4me3 markers, which mark gene as transcriptionally active [28], enabling constant production of the proteins demanded by the core functions of life. For those temporally and spatially expressed pluripotency genes demanded during development [29], recent research has demonstrated that H3K27me3 blocks the activation of key regulators of embryonic development [30]. Co-occupancy of histone modifications may provide a mechanism to switch genes on or off swiftly in different cell types and developmental stages.

### Gene age preferences of histone modifications in early embryos

Previously, we observed that during DNA methylome reprogramming in zebrafish and mouse, evolutionarily young genes tend to undergo extensive reprogramming compared to older ones (unpublished data). We asked whether there are preferences in gene age in the context of histone modification states, as new genes evolved to render novel functions during species evolution. To address this question, a phylostratigraphic approach [31] was used. In this method, protein coding genes of zebrafish and mouse were assigned to a certain phylostratum which represented the gene age [32]. By comparing the proportion of genes assigned to a certain phylostratum in selected gene groups to the proportion in the whole genome, we could find over- and under-represented phylostratum in each group.

For the co-occupied gene group in zebrafish (Fig. 4c), the proportion of phylostrata 4 and 5 was significantly higher than that in the whole genome, while the proportions of the first two and last two phylostrata were significantly lower. In mouse, we find that the proportions of phylostrata 3–5 were higher and the proportions of phylostrata 1, 6, 12, 16, and 19 were lower than those in the whole genome (Fig. 4e). Genes originating from organisms that evolved during phylostratum 4 (opisthokonta to holozoa) and phylostratum 5 (holozoa to metazoa), which are multicellular species, are more likely to contain co-occupied modifications in both mouse and zebrafish.

For the active gene groups, genes originating in primitive unicellular organisms (phylostratum 1) and eukaryotic organisms (phylostratum 2) were significantly enriched. The proportion of genes that diverged from holozoa organism onward (phylostrata 5–14) are significantly lower in zebrafish (Fig. 4d). In mouse, which is similar to zebrafish, genes from first three phylostrata, which are the oldest genes, are more likely to contain active marks than the younger genes (phylostratum 5 onward) (Fig. 4f). In contrast to co-occupied and active gene groups, gene groups B, D and E contain more young genes. The gene age preferences for histone modification status were also confirmed in each stage (Additional file 8: Table S7and Additional file 9: Table S8). We further examined the gene age preferences for the active and co-occupied gene groups as well as all genes in each stage and found that opisthokonta, in which multicellular organisms originated, is the key boundary for gene age preference. Genes that diverged before opisthokonts are more likely to be favored by active modifications, while genes that arose between opisthokonta and metazoa are preferred by co-occupied histone modifications. In summary, gene age preference reflects the phylotypic phase [31] during the transition from unicellular to multicellular organisms.

## Discussion

We present the histone modification dynamics in the early embryos of zebrafish and draw comparisons with those in the early embryos of mouse. As embryos develop, repressive markers are erased, and states of histone modification are stabilized upon ZGA. It has been reported that H3K4me3 enrichments in paternal genome are first depleted and then reestablished in early mouse embryos [12]. In our data, we also observed that H3K4me3 enrichments for active gene group are first erased and then approximately restored to match the gamete pattern upon ZGA. Compared to mouse, the prolonged ZGA duration of the zebrafish embryo offers great opportunities to observe detailed reprogramming processes for co-occupied genes. This analysis would require much more effort in early mouse embryos. We summarized that reprogramming procedures are overall conserved between species differ in details.

In another aspect, we observed that histone modification reprogramming does not reset the entire pattern to match the pattern of either gamete as DNA methylation dose in early embryos. Furthermore, hypermethylated promoter peaks are found to mark stage-specific genes as DNA methylation levels decreased uniformly in both distal and promoter peaks in mouse. In zebrafish, this is true for promoter peaks but not for distal peaks. Combining DNA methylation and histone modification patterns in early embryos and other cell lineages [30, 33], we reasoned that histone modifications are potentially reprogrammed independently of DNA methylation.

Histone modification status is ready for gene expression prior to ZGA. The two major groups of genes more likely to be modified arose from different phylostrata. Evolutionary gene ages were distinguished by considering genes as evolving before or after opisthokonts, which are commonly characterized with flagellate cells. Opisthokonts are a broad group of eukaryotes, included both the animal and fungus kingdoms, and are thought to have evolved from protist ancestors [34]. Developmental complexity increased for species within opisthokonta as diverse cell types with different functions differentiated. Novel genes that diverged to regulate cell differentiation, providing sophisticated biological functions, are required to be precisely regulated in their gene regulatory network (GRN). Hence, gene age preferences reflect the phylotypic phase during the transitions from unicellular to multicellular organisms. Despite the core genes, other regulatory mechanisms, such as non-coding RNA and alternative transcript isoforms, also shape the fate of mRNA under environmental pressures, such as enhancing or reducing translation efficiency. We also observed that stage specific modification enrichments, which generally appeared around young genes, are rapidly discarded during early development while old genes are more likely to be constantly active or co-occupied. According to our observation, it is possible that novel or young genes are firstly added to a specific GRN by being lowly expressed, and then being more expressed when the functional role results in a “good” phenotype. This is the part we do not described but should be interesting in future investigations.

While this manuscript was being prepared, Muprhy et al. [35] reported that placeholder nucleosomes mark housekeeping and early embryonic vital factors in early embryo before genome activation. And while our manuscript being reviewed, Zhang et al. [36] also described the reprogramming process of H3K4me3 in early zebrafish embryo and suggested that histone states are ready for transcription prior to ZGA. Our observations are in line with these recently published results.

## Conclusions

The transitions from unicellular to multicellular organisms are key events in the evolution of life. Multicellular organisms require precise regulation of diverse cell types to fulfill developmental requirements. Epigenetic modifications, such as DNA modifications and histone modifications, could be utilized to achieve that purpose. The question of how epigenetic modifications are reprogrammed and inherited in early embryos has existed for a long time and has been answered thoroughly for DNA methylation. However, understanding of histone modifications is limited. In mouse, noncanonical broad H3K4me3 domains have been reported in oocytes, but this feature does not exist in zebrafish oocytes due to the lack of partially methylated domains (PMD). In our study, we profiled histone modifications of early embryos in zebrafish and revealed that there is extensive reprogramming of histone modifications. Compared with the reprogramming process in mouse, we found that although there are differences in histone modification reprogramming, there are conserved principles followed in both zebrafish and mouse. Our data suggested that the reprogramming of histone modifications is likely independent of reprogramming DNA methylation. Histone modification patterns passed on from gametes are reprogrammed according to their associated genes’ functions, thereby facilitating maternal-to-zygotic gene expression transitions and embryogenesis.

## Methods

### Zebrafish stock

Wild-type zebrafish line TU, maintained in our own laboratory, was used in this study. Zebrafish were raised for three to twelve months under standard conditions (28 °C, 14 h light / 10 h darkness) before experimental materials were collected.

### Zebrafish sperm collection

Adult male fish were first anesthetized in system water containing 0.016% Tricaine. Testes were dissected from adult male fish as described [37] and placed in 10 ml PBS buffer (filtered by 0.22um filter) for 10 min at room temperature. During that time, the testes were pipetted several times. Tissue fractions were removed with 75 nm filter units before they were transferred to 15 ml tubes. The tubes were centrifuged for 1 min at 300 g and then held still for 3 min. After that, the upper half of the liquid was transferred to a new 10 ml tube, which was filled with PBS to 10 ml. The last step was repeated again. The sperm purity was checked under a microscope, and additional steps were performed if the purity was not sufficient. Aliquot of approximately 1 million sperm per tube were made for one ChIP experiment. The tubes were centrifuged for 10 min at 10,000 g, and the supernatant was discarded. Cross-linking or snap freezing of the sperm sample in liquid nitrogen was performed before storage at − 70 °C.

### Oocyte and early embryo collection

Approximately 5 min after spawning three washes with system water, unfertilized oocytes were collected by squeezing the abdomen of females. The squeezed females were transferred to separate tanks for recovery. Embryos were collected for 10–15 min after each round of spawning and washed with system water three times. Collected unfertilized oocytes and embryos were washed three times with system water to remove potential follicle cells or other contaminants and then were transferred to 90 mm glass petri dishes with approximately 10 ml system water before proceeding to next step. Embryos were grown at 28 °C and staged according to standard morphological criteria [38], Three minutes prior to the desired embryonic stage or immediately after the collection of oocytes, prewarmed (approximately 28 °C) pronase solution (2 mg/ml at final concentration) was added to oocytes and embryos to perform dechorionation. The dishes were placed on ice to prevent embryos and oocytes from developing further, the chorion was disrupted with a glass dropper, and then the embryos were washed three times with cold (4 °C) HBSS buffer to remove chorion fragments. One thousand embryos or oocytes (for one ChIP experiment) were transferred to 1.5 ml low-binding tubes with a glass dropper. One ml of cold (4 °C) deyolk buffer [18] were add to each tube after removing the HBSS buffer, and then the tube was rotated for 3 min at 4 °C. After centrifugation for 3 min at 1000 g, the supernatant was discarded. We proceeded directly to cross-linking steps or snap freezing the samples in liquid nitrogen before storage at − 70 °C. After collecting eggs and embryos, fish were raised in separated tanks until full recovery.

### Cross linking

Tubes with pellets were filled with cold PBS buffer to 1 ml, 37% formaldehyde solution (sigma) was added to a final concentration of 1%, and then tubes were rotated for 10 min at room temperature. Cross linking was halted by adding fresh glycine solution to a final concentration of 125 mM, and tubes were rotated for another 5 min. The cross-linked cells were centrifuged for 10 min at 10,000 g and washed two times with cold PBS buffer. The supernatant was removed, and cell pellets were stored at − 70 °C or directly used in chromatin immunoprecipitation steps.

### Chromatin immunoprecipitation and library preparation

The bead-antibody complexes were prepared before cell lysis using 2 μg of H3K4me3 (Abcam ab8580) antibody or 2.5 μg of H3K27me3 (Millipore 07–449) antibody per 10 μl of protein A Dynabeads in low binding PCR tubes. Beads were washed two times with 100 μl ChIP buffer (10 mM Tris-HCl, pH 7.5; 1 mM EDTA; 0.5 mM EGTA; 0.1% Sodium Deoxycholate; 1% Triton X-100; 0.1% SDS; 140 mM NaCl; fresh proteinase inhibitor cocktail), then the total volume was brought up to 100 μl per assay. The bead-antibody complexes were incubated on a rotator at 4 °C for 2 h. The ChIP buffer was removed before adding the sonicated chromatin. Cell were lysed with 100 μl of lysis buffer (0.2% SDS; 10 mM Tris-HCl buffer, pH 8.0; 10 mM EDTA, pH 8.0; fresh proteinase inhibitor cocktail) on ice for 30 min. Lysed cells were sonicated in Bioruptor for 25 cycles (30s on and 30 s off per cycle) to achieve fragments that were 300–500 bp in length. The tubes were centrifuged for 10 min at 4 °C (> 10,000 rpm), and 100 μl of supernatant was transferred to the bead-antibody complex tubes. The tubes were rotated slowly at 4 °C overnight (8–10 h). The next day, the tubes were placed on a magnetic rack for one minute, and the supernatant was removed. The beads were washed two times with low salt buffer (10 mM Tris-HCl, pH 8.0; 1 mM EDTA; 0.5 mM EGTA; 1% Triton X-100; 0.1% SDS; 250 mM NaCl) and high salt buffer (10 mM Tris-HCl, pH 8.0; 1 mM EDTA; 0.5 mM EGTA; 1% Triton X-100; 0.1% SDS; 500 mM NaCl); one time with LiCl buffer (10 mM Tris-HCl, pH 8.0; 1 mM EDTA; 0.5 mM EGTA; 1% Triton X-100; 0.1% SDS; 300 mM LiCl). The supernatant was removed, and TE buffer was added (20 mM Tris-HCl, pH 8.0; 10 mM EDTA, pH 8.0) before the beads were transferred to another new tube. The supernatant was discarded, and 100 μl of elution buffer was added (20 mM Tris-HCl, pH 8.0; 5 mM EDTA, pH 8.0; 50 mM NaCl; 1% SDS; 2 mg/ml proteinase K). The tubes were placed on a thermal shaker operated at 68 °C and 1000 rpm for 6–8 h. DNA fragments were purified with AMPure XP beads (Beckman) directly before library preparation following Illumina’s instructions. For each sampled stage, two ChIP replicates were performed, and libraries were prepared individually for each replicate except for H3K27me3 ChIP with oocytes. For the H3K27me3 ChIP assay performed in oocytes, precipitated DNA fragments were pooled from two replicates to provide enough DNA for library preparation.

### Date processing: read mapping, peak calling and annotations, visualization

ChIP-Seq data generated in this study or public ChIP-Seq data (dome and 48 hpf stages) used in this study were processed as following. Low quality and adapter-containing reads were trimmed using Trimmomatic [39]. High-quality reads then were fed into Bowtie2 [40] for mapping against the zebrafish genome (Zv9). Uniquely mapped read pairs were kept for downstream analysis. Duplicated reads were removed with Picard tools (http://broadinstitute.github.io/picard). The replicates of each stage were merged with equivalent read pairs and peaks were called using MACS2 [41] with default settings (except that ‘--broad’ was specified) and filtered with a qValue cutoff set to 0.05. For mouse, data for called peaks were directly downloaded from the NCBI GEO database. Peak annotations were done using the R package ChiPpeakAnno [42] against Ensembl gene annotations for zebrafish genome Zv9 or mouse genome mm9, assigning each peak to the nearest transcription start site. Those peaks that fell between 3 kb upstream and 3 kb downstream of the TSS were kept for further analysis. For data visualization, we adjust ChIP-seq data with internal reference to account for enrichment fold differences among stages. We choose peak that overlap with promoter of eef1a1b, which is a translation elongation factor that is reported stable in various developmental stages [43]. ChIP-seq track screenshots are plotted with Gviz [44] package in R environment.

### GO, INTERPRO and gene Phylostratum enrichment analysis

Gene ontology and INTERPRO domain enrichment analyses were carried out using the DAVID web service [45, 46] with annotated Ensemble gene IDs. The enriched items were filtered with the FDR cutoff set to 0.01. Gene phylostratum information was acquired from previously published data [32], and enrichment analysis was performed with a hypergeometric test in R with the FDR cutoff set to 0.01 for determining significantly enriched phylostrata in each gene group.

### Statistics test for significance

The Wilcox test was used in this study to determine the significant differences between groups unless otherwise specified in the manuscript. The significance level was uniformly set to 0.01.

## Additional files

Additional file 1: **Figure S1.** Data quality assessments. **a** Pearson correlation of genome wide coverage between replicates. For H3K27me3 ChIP assay in oocyte, we pooled all ChIP DNA from two replicates as DNA from single replicate is not adequate for library construction. **b** Overlaps of marked genes between our data and previous ChIP-chip results. **c** Comparison of proportion of H3K27me3 marked genes in 1 K stage that are covalent with H3K4me3 between our data and previous ChIP-chip results. **Note**:**‘**n.s.’indicates there is no significant difference in proportion of genes according to Chi-Square test. **Figure S2.** Differences of peak width in distal and promoter peak for H3K4me3 and H3K27me3. **a** Width of promoter peaks is significantly larger than it in distal peaks for H3K4me3. **b** Width of promoter peaks is significantly larger than it in distal peaks for H3K27me3. ‘***’ denotes that there is significant difference by Wilcox test (*p* < 0.01). **Figure S3.** Differencesof CpG dinucleotidedensity in distal and promoter peak for H3K4me3 and H3K27me3. **a** CpG dinucleotidedensity of promoter peaks is significantly larger than it in distal peaks for H3K4me3. **b** CpG dinucleotidedensity of promoter peaks is significantly larger than it in distal peaks for H3K27me3. ‘***’ denotes that there is significant difference by Wilcox test(p < 0.01); ‘n.s.’ means there is no significant difference by Wilcox test (*p* > 0.01). **Figure S4.** Examples of genes showing histone modification reprogramming patterns during ZGA. **Figure S5.** Histone modification landscapes of *hox*gene cluster in zebrafish. **Figure S6.** Histone modifications dynamics in mouse. **a** Histone modification dynamics for genes in mouse. **b** Hierarchy clustering of sampled developing stages. **Figure S7.** Correlation between RNA abundanceand histonemodification enrichment. **a** RNA-Seq FPKM is positively correlated with H3K4me3 FPKMand it increased as embryo developing. **b** RNA-Seq FPKM is negatively correlated with H3K27me3 FPKM. **Figure S8.** Relationship between DNA methylation and histone modification enrichment. **a** DNA methylation level and histone modification enrichment are negatively associated in promoter regions (TSS +/− 3 kb) for both H3K4me3 and H3K27me3. **b** Histone modification and DNA methylation landscapes of *dnmt*gene family. **Figure S9.**. DNA methylation levels in distal and promoter peak regions are significantly different in mouse. Note, ‘***’ denotes there is significant difference between group with Wilcox test (p < 0.01). **Figure S10.** Functional conservation in bivalent and active gene groups between zebrafish and mouse. **a** Conserved INTERPRO domains between zebrafish and mouse for bivalent gene group. **b** Conserved GO terms between zebrafish and mouse for bivalent gene group. **c** Conserved INTERPRO domains between zebrafish and mouse for active gene group. **d** Conserved GO terms between zebrafish and mouse for active gene group. (PDF 2365 kb)

Additional file 2: **Table S1.** Pairwise dissimilarities for each stage in zebrafish (XLSX 9 kb)

Additional file 3: **Table S2.** Pairwise dissimilarities for each stage in mouse (XLSX 9 kb)

Additional file 4: **Table S3.** Enriched GO and INTERPRO domains for co-occupied gene group in zebrafish. (XLSX 11 kb)

Additional file 5: **Table S4.** Enriched GO and INTERPRO domains for active gene group in zebrafish. (XLSX 15 kb)

Additional file 6: **Table S5.** Enriched GO and INTERPRO domains for co-occupied gene group in mouse. (XLSX 11 kb)

Additional file 7: **Table S6.** Enriched GO and INTERPRO domains for active gene group in mouse. (XLSX 15 kb)

Additional file 8 **Table S7** Gene age preference for each modification status in each stage of zebrafish. (XLSX 12 kb)

Additional file 9: **Table S8.** Gene age preference for each modification status in each stage of mouse. (XLSX 11 kb)

## Abbreviations

ChIP: Chromatin Immunoprecipitation
ChIP-Seq: Chromatin Immunoprecipitation Sequencing
PS: Phylostratum
ZGA: Zygotic Genome Activation
