# Supplementary material for: Reprogramming histone modification patterns to coordinate gene expression in early zebrafish embryos
Source: BMC Genomics. 2019 Mar 28;20:248. doi: 10.1186/s12864-019-5611-7 (PMC6437866; doi:10.1186/s12864-019-5611-7)
Supplement: Supplementary file 1 — Figure S1. Data quality assessments. a Pearson correlation of genome wide coverage between replicates. For H3K27me3 ChIP assay in oocyte, we pooled all ChIP DNA from two replicates as DNA from single replicate is not adequate for library construction. b Overlaps of marked genes between our data and previous ChIP-chip results. c Comparison of proportion of H3K27me3 marked genes in 1 K stage that are covalent with H3K4me3 between our data and previous ChIP-chip results. Note:‘n.s.’indicates there is no significant difference in proportion of genes according to Chi-Square test. Figure S2. Differences of peak width in distal and promoter peak for H3K4me3 and H3K27me3. a Width of promoter peaks is significantly larger than it in distal peaks for H3K4me3. b Width of promoter peaks is significantly larger than it in distal peaks for H3K27me3. ‘***’ denotes that there is significant difference by Wilcox test (p < 0.01). Figure S3. Differencesof CpG dinucleotidedensity in distal and promoter peak for H3K4me3 and H3K27me3. a CpG dinucleotidedensity of promoter peaks is significantly larger than it in distal peaks for H3K4me3. b CpG dinucleotidedensity of promoter peaks is significantly larger than it in distal peaks for H3K27me3. ‘***’ denotes that there is significant difference by Wilcox test(p < 0.01); ‘n.s.’ means there is no significant difference by Wilcox test (p > 0.01). Figure S4. Examples of genes showing histone modification reprogramming patterns during ZGA. Figure S5. Histone modification landscapes of hoxgene cluster in zebrafish. Figure S6. Histone modifications dynamics in mouse. a Histone modification dynamics for genes in mouse. b Hierarchy clustering of sampled developing stages. Figure S7. Correlation between RNA abundanceand histonemodification enrichment. a RNA-Seq FPKM is positively correlated with H3K4me3 FPKMand it increased as embryo developing. b RNA-Seq FPKM is negatively correlated with H3K27me3 FPKM. Figure S8. Relationship between DNA methylatio [file 12864_2019_5611_MOESM1_ESM.pdf]

Supplementary Figures

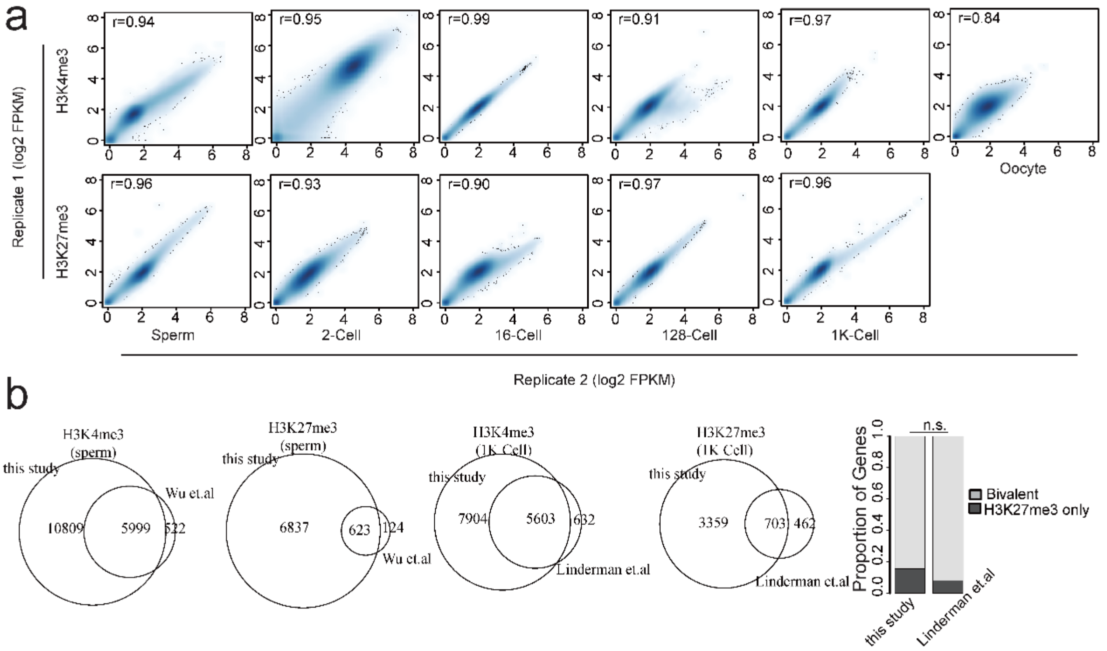

Figure S1. Data quality assessments. **a** Pearson correlation of genome wide coverage between replicates. For H3K27me3 ChIP assay in oocyte, we pooled all ChIP DNA from two replicates as DNA from single replicate is not adequate for library construction. **b** Overlaps of marked genes between our data and previous ChIP-chip results. **c** Comparison of proportion of H3K27me3 marked genes in 1K stage that are covalent with H3K4me3 between our data and previous ChIP-chip results.

Note: **'**n.s.' indicates there is no significant difference in proportion of genes according to Chi-Square test.

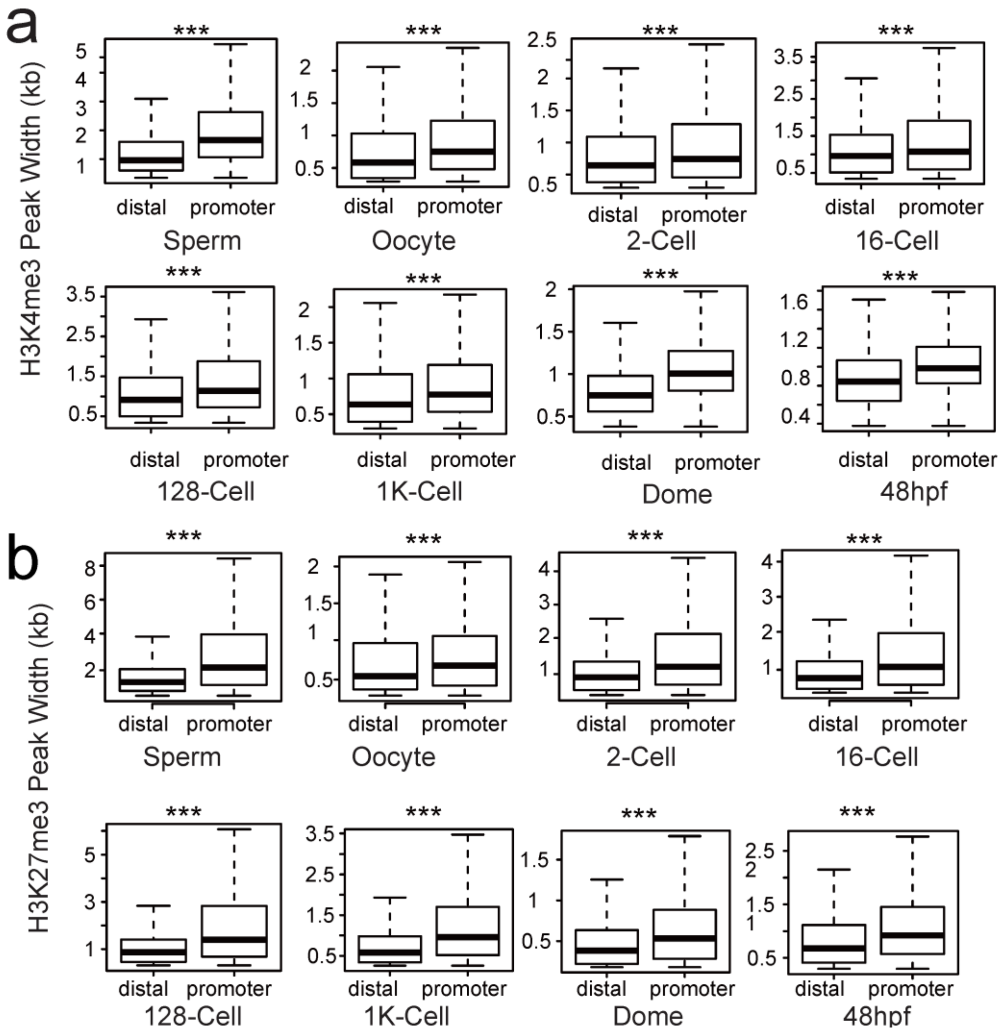

Figure S2. Differences of peak width in distal and promoter peak for H3K4me3 and H3K27me3. **a** Width of promoter peaks is significantly larger than it in distal peaks for H3K4me3. **b** Width of promoter peaks is significantly larger than it in distal peaks for H3K27me3. **'***'** denotes that there is significant difference by Wilcox test ($p<0.01$).

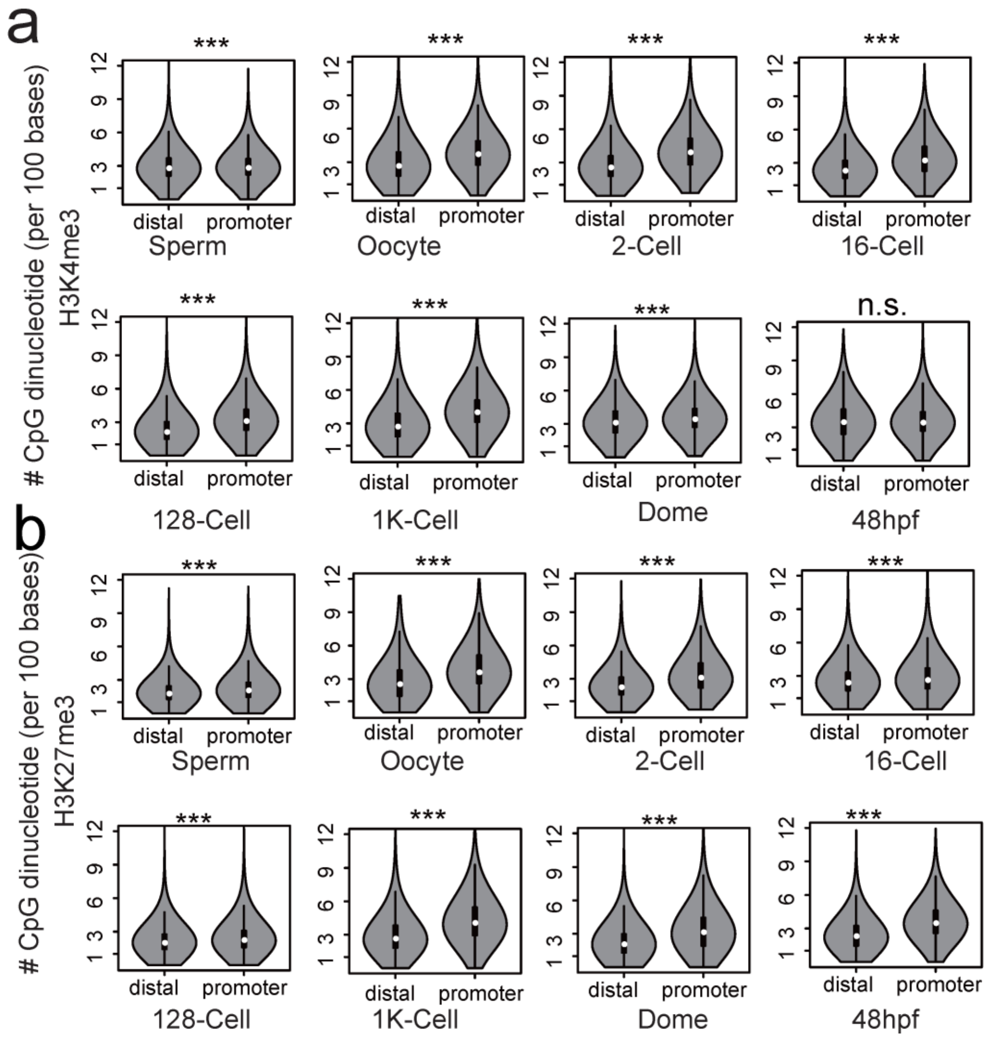

Figure S3. Differences of CpG dinucleotide density in distal and promoter peak for H3K4me3 and H3K27me3. **a** CpG dinucleotide density of promoter peaks is significantly larger than it in distal peaks for H3K4me3. **b** CpG dinucleotide density of promoter peaks is significantly larger than it in distal peaks for H3K27me3. **'***'** denotes that there is significant difference by Wilcox test ($p<0.01$); 'n.s.' means there is no significant difference by Wilcox test ($p>0.01$).

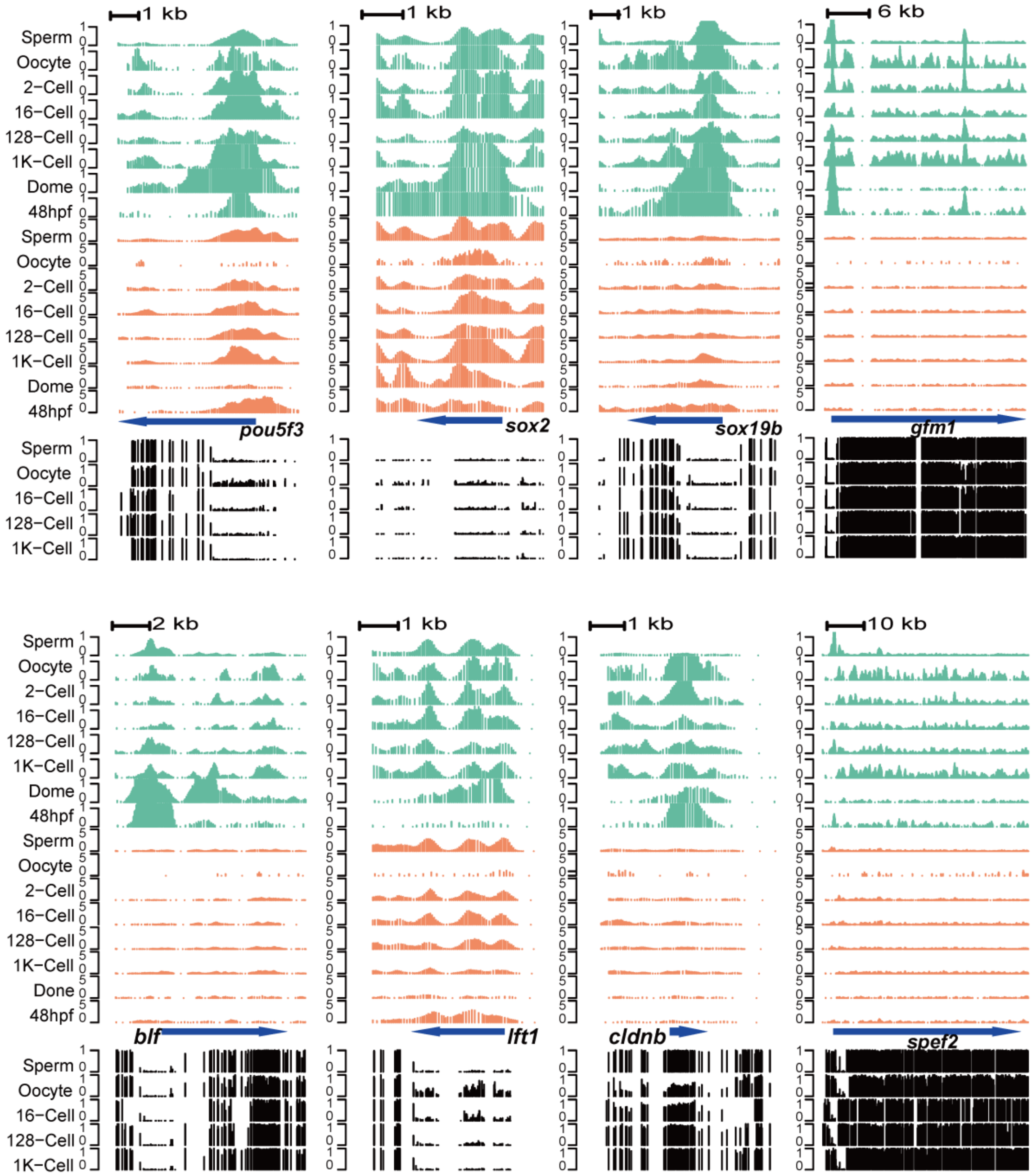

Figure S4. Examples of genes showing histone modification reprogramming patterns during ZGA.

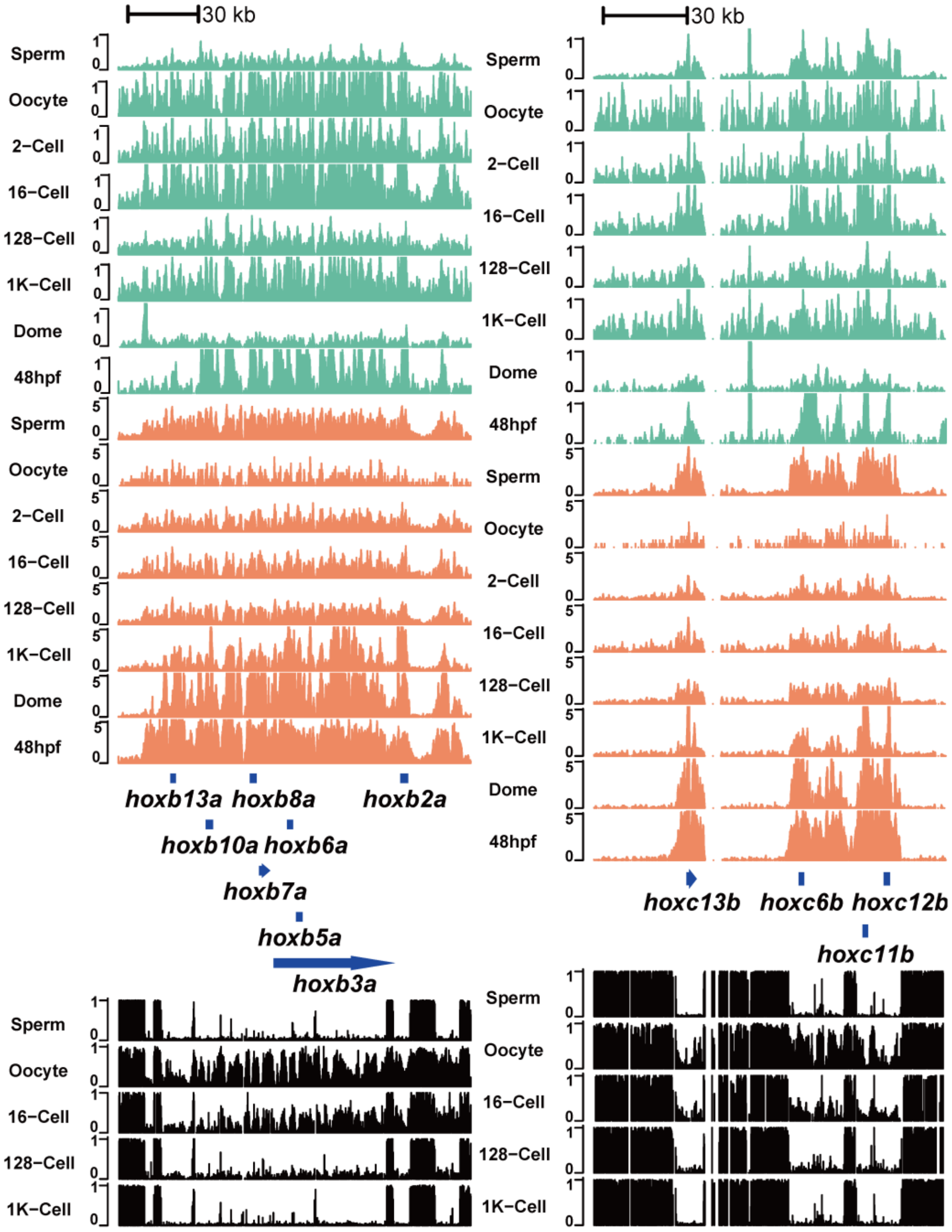

Figure S5. Histone modification landscapes of *hox* gene cluster in zebrafish.

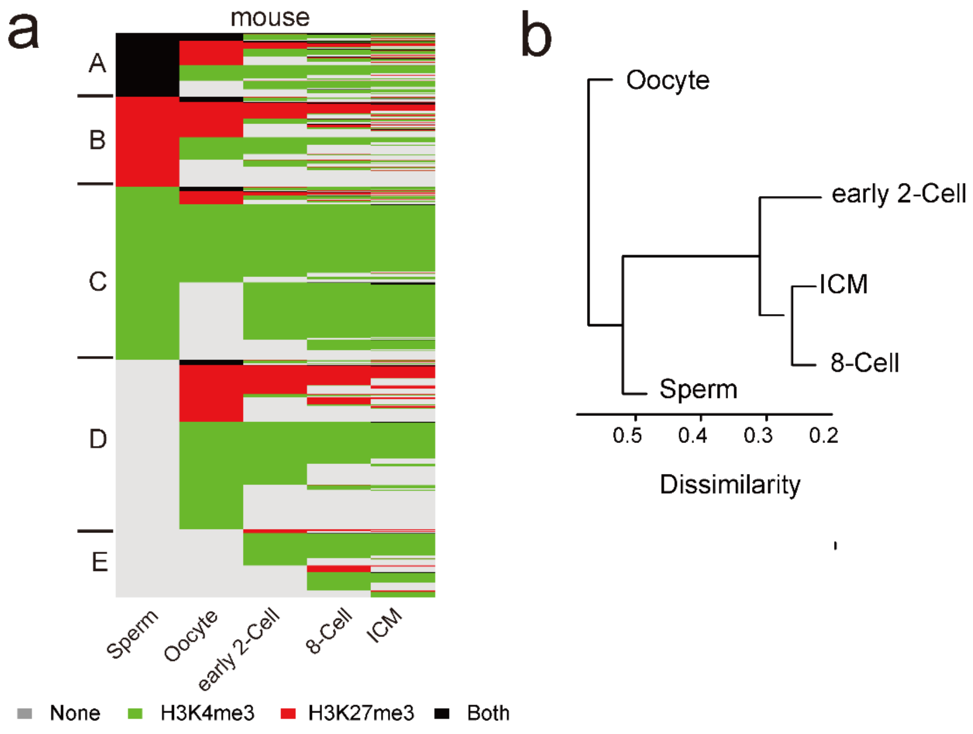

Figure S6. Histone modifications dynamics in mouse. **a** Histone modification dynamics for genes in mouse. **b** Hierarchy clustering of sampled developing stages.

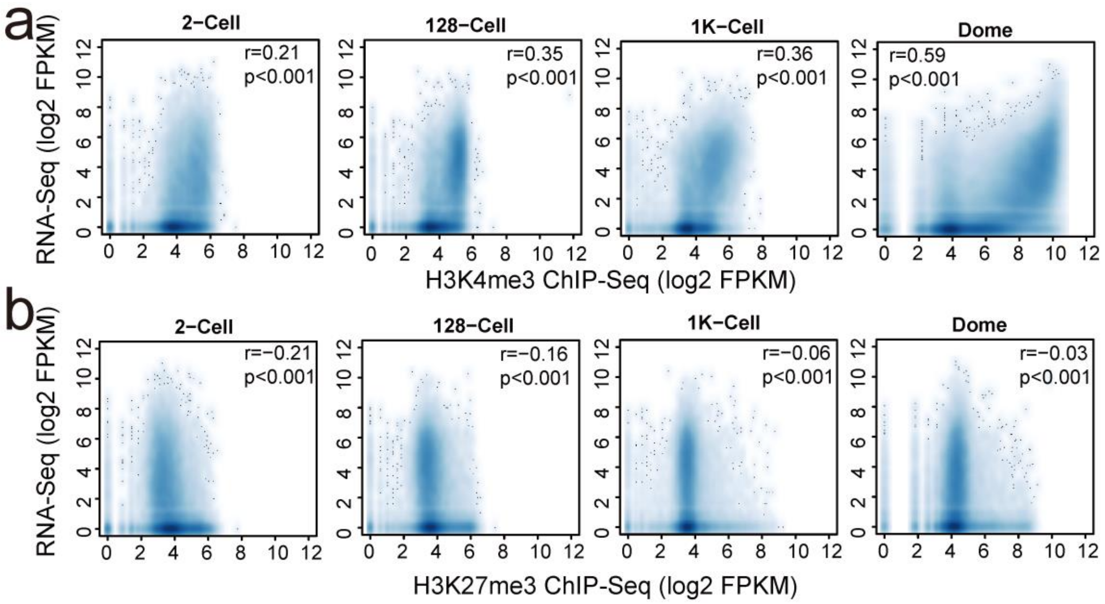

Figure S7. Correlation between RNA abundance and histone modification enrichment. **a** RNA-Seq FPKM is positively correlated with H3K4me3 FPKM and it increased as embryo developing. **b** RNA-Seq FPKM is negatively correlated with H3K27me3 FPKM.

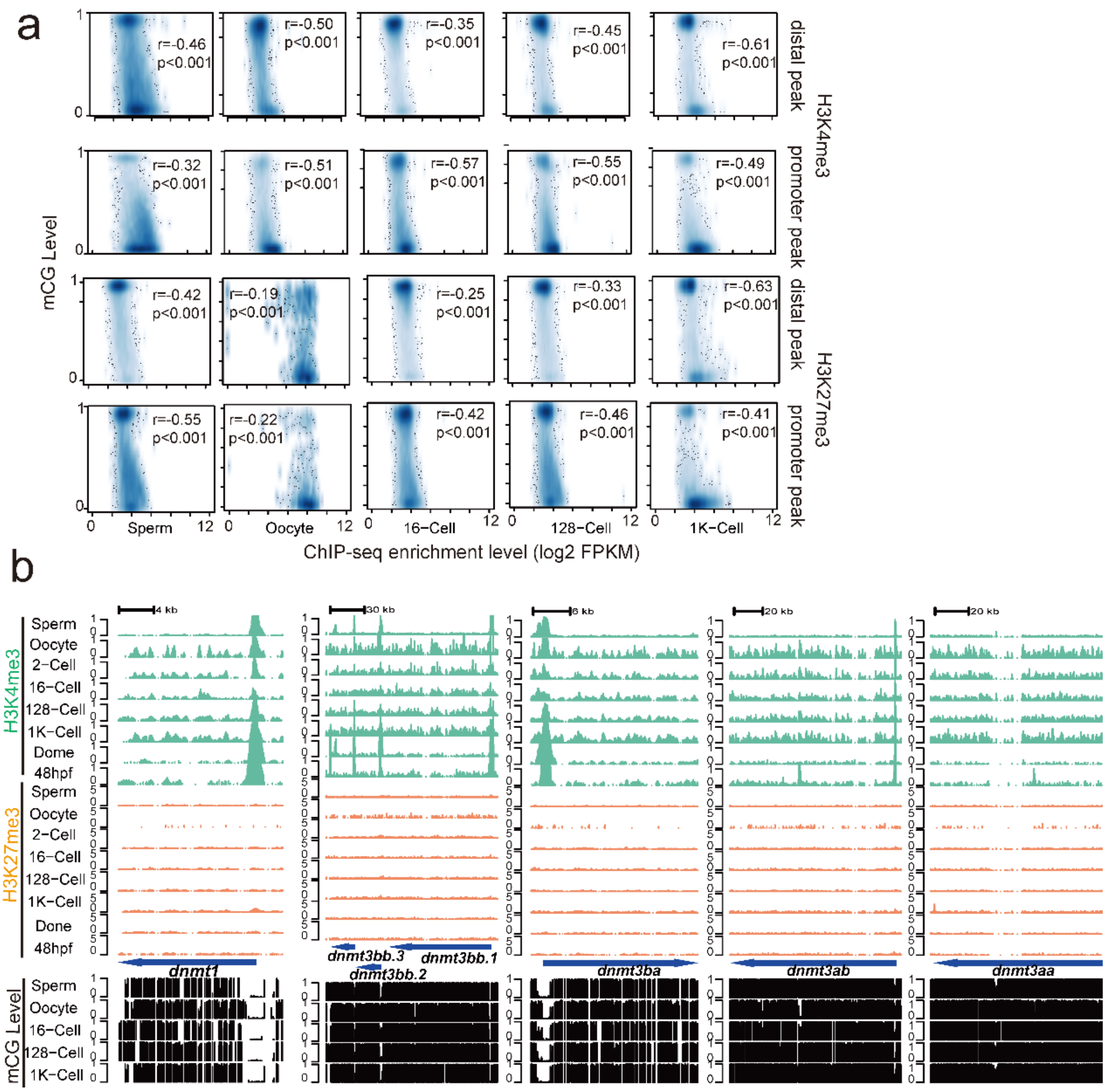

Figure S8. Relationship between DNA methylation and histone modification enrichment. **a** DNA methylation level and histone modification enrichment are negatively associated in promoter regions (TSS +/- 3 kb) for both H3K4me3 and H3K27me3. **b** Histone modification and DNA methylation landscapes of *dnmt* gene family.

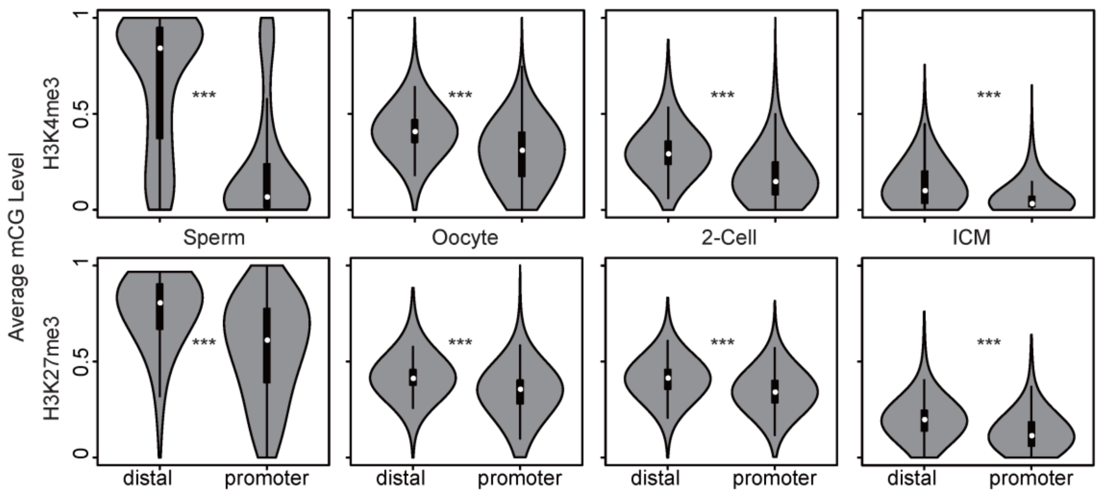

Figure S9. DNA methylation levels in distal and promoter peak regions are significantly different in mouse. Note, '***' denotes there is significant difference between group with Wilcox test (p<0.01).

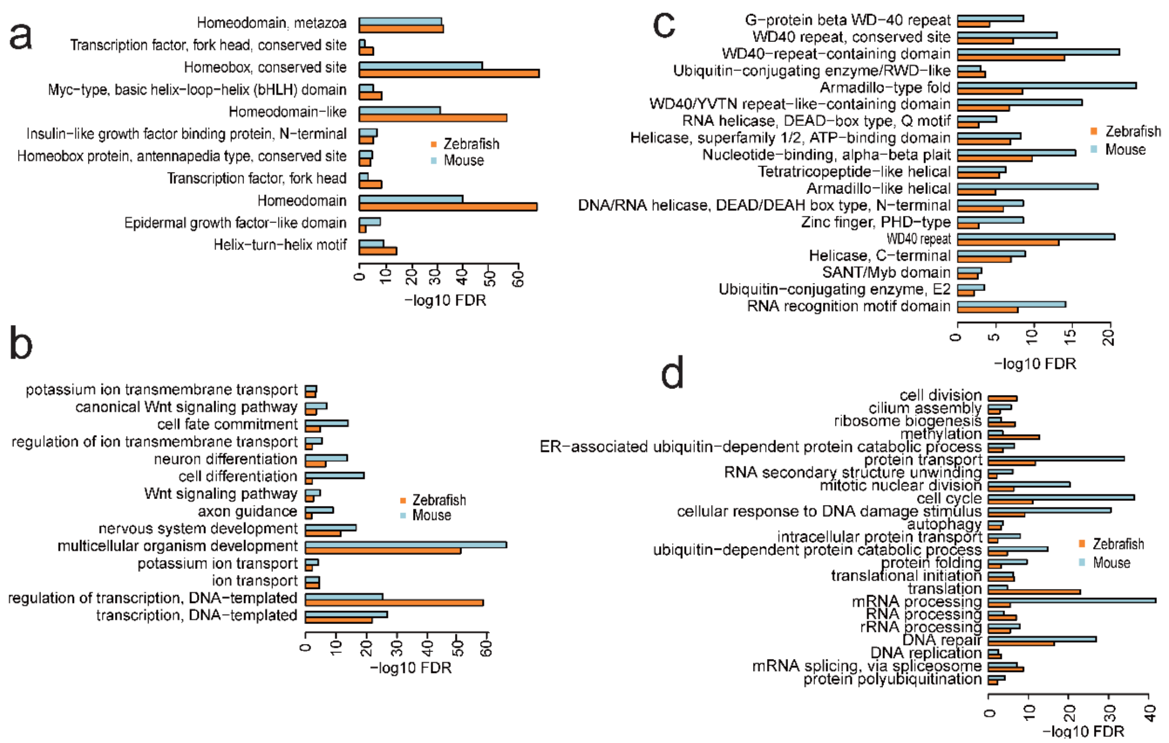

Figure S10. Functional conservation in bivalent and active gene groups between zebrafish and mouse. **a** Conserved INTERPRO domains between zebrafish and mouse for bivalent gene group. **b** Conserved GO terms between zebrafish and mouse for bivalent gene group. **c** Conserved INTERPRO domains between zebrafish and mouse for active gene group. **d** Conserved GO terms between zebrafish and mouse for active gene group.
